# Pupillometric investigation into the speed‐accuracy trade‐off in a visuo‐motor aiming task

**DOI:** 10.1111/psyp.13499

**Published:** 2019-11-17

**Authors:** Marnix Naber, Peter Murphy

**Affiliations:** ^1^ Experimental Psychology Helmholtz Institute, Utrecht University Utrecht The Netherlands; ^2^ Vision Sciences Laboratory Harvard University Cambridge Massachusetts; ^3^ Section Computational Cognitive Neuroscience, Department of Neurophysiology and Pathophysiology University Medical Center Hamburg‐Eppendorf Hamburg Germany

**Keywords:** motor control, motoric aiming, pupil size, pupillometry, speed‐accuracy trade‐off

## Abstract

Convergent lines of evidence suggest that fluctuations in the size of the pupil may be associated with the trade‐off between the speed (adrenergic, sympathetic) and accuracy (cholinergic, parasympathetic) of behavior across a variety of task contexts. Here, we explored whether pupil size was related to this trade‐off during a visuospatial motor aiming task. Participants were shown visual targets at random locations on a screen and were instructed and incentivized to move a computer mouse‐controlled cursor to the center of the targets, either as fast as possible, as accurately as possible, or to strike a balance between the two. Behavioral results showed that these instructions led to typical speed‐accuracy trade‐off effects on movement reaction times and hit distances to target centers. Pupillometric analyses revealed that movements were faster and less accurate when participants had relatively large baseline pupil sizes, as measured before target onset. Furthermore, trial‐evoked pupil dilation was related specifically to a bias toward speed in the trade‐off and the speed of the ballistic and error‐correction phases of the motor responses such that larger pupils predicted shorter latencies and higher movement speeds. Pupil responses were also associated with performance in a manner that may reflect the combined influence of a number of factors, including the generation of dynamic urgency and an arousal response to negative feedback. Our results generally support a role for pupil‐linked arousal in regulating the trade‐off between speed and accuracy, while also highlighting how the trial‐related pupil response can exhibit multifaceted, temporally discrete associations with behavior.

## Abstract 2

The eye’s pupil has been considered a “window into the soul” as its dynamics are related to a wide variety of cognitive processes. Here, we present convergent evidence that both slow, prestimulus fluctuations and fast, event‐related changes in pupil diameter are sensitive to a fundamental trade‐off between the speed and accuracy of visuo‐motor actions—an association that holds for both instructed and endogenous variation in this trade‐off. This finding complements a growing literature linking pupil size to adaptive, contextually appropriate changes in behavior.

## 1 INTRODUCTION

The pupil is a hole in the iris of the eye that can change in size. Just like the diaphragm of a camera lens, its main ocular function is to regulate the visual input admitted to the eye's retina. In so doing, the pupil controls two ocular effects: exposure (i.e., the amount of light hitting the retina) and the depth of field (i.e., whether objects within a small vs. large depth plane are sharply focused on the retina). Pupil size is determined by two sets of muscles in the iris: the parasympathetic, cholinergically innervated sphincter muscle constricts the pupil, while the sympathetic, noradrenergically innervated dilator muscle dilates the pupil. Thus, the size of the pupil at any one time is affected by the push‐pull dynamics of opposing arms of the autonomic nervous system (Beatty & Lucero‐Wagoner, 2000; Koss, 1986).

It is well known that the size of the pupil adapts to the level of ambient light in the environment. Yet, pupil size also fluctuates significantly when luminance remains constant, and decades of research have related such nonluminance‐mediated pupil changes to aspects of cognition. Initial reports established links between dilation of the pupil and the “cognitive effort” expended during performance of a given task (Beatty, 1982), for example, when solving mathematical problems of varying difficulty (Hess & Polt, 1964). Since then, changes in pupil size have been related to many more cognitive operations, including perception (Einhäuser, Stout, Koch, & Carter, 2008; Naber & Nakayama, 2013; Wierda, van Rijn, Taatgen, & Martens, 2012), attention (Binda, Pereverzeva, & Murray, 2013; Mathôt, Van der Linden, Grainger, & Vitu, 2013; Naber, Alvarez, & Nakayama, 2013), decision making (de Gee, Knapen, & Donner, 2014; Gilzenrat, Nieuwenhuis, Jepma, & Cohen, 2010; Jepma & Nieuwenhuis, 2011; Murphy, Vandekerckhove, & Nieuwenhuis, 2014), learning (Krishnamurthy, Nassar, Sarode, & Gold, 2017; Nassar et al., 2012), memory encoding (Naber, Frässle, Rutishauser, & Einhäuser, 2013; Sterpenich et al., 2006), conflict/error monitoring (Ebitz & Platt, 2015; Murphy, Boonstra, & Nieuwenhuis, 2016; Murphy, van Moort, & Nieuwenhuis, 2016; Van Steenbergen & Band, 2013), and consciousness (Fahle, Stemmler, & Spang, 2011; Naber, Frässle, & Einhäuser, 2011). These studies have variously established relationships between cognition and fluctuations in pupil size that take place over two timescales: slow “tonic” fluctuations that are typically assessed by measuring pupil diameter in the pretrial period and comparatively fast “phasic” pupil dilations and constrictions that are evoked by presentation of task‐related stimuli.

Here, we characterize the sensitivity of pupil fluctuations measured over both timescales to the speed and accuracy of responses during simple visuo‐motor aiming behavior. A visuo‐motor aiming task requires movements toward a target and can therefore probe response speed and accuracy, which depend on the spatial properties of the target within a single trial.

Two lines of argument suggest that pupil size might exhibit a general relationship to the speed‐accuracy trade‐off that is not limited to any one task context. First, it is an established finding that the latencies of both neural responses and motor reactions to bright objects are shorter than those to dark objects (e.g., Vaughan, Costa, & Gilden, 1966). Given the essential role of the pupil in controlling retinal illumination and thus the perceived brightness of visual stimuli, it follows that larger pupil sizes may be associated with faster responses. Conversely, pupil constriction leads to a broader depth of field, which increases the likelihood that objects are perceived in sharp focus. Although this effect is small and nonlinear (Woodhouse & Campbell, 1975), it implies that smaller pupils may in some cases be associated with more accurate visual representations (but see Warren et al., 2016). These considerations suggest that fluctuations in pupil size potentially exert a causal influence on the speed and accuracy of visual processing.

Second, pupil size might be indirectly associated with the speed and accuracy of behavior via its covariation with the activity of certain structures in the brain. Recent studies in humans (de Gee et al., 2017; Murphy, O'Connell, O'Sullivan, Robertson, & Balsters, 2014), monkeys (Joshi, Li, Kalwani, & Gold, 2016; Varazzani, San‐Galli, Gilardeau, & Bouret, 2015), and rodents (Breton‐Provencher & Sur, 2019; Liu, Rodenkirch, Moskowitz, Schriver, & Wang, 2017; McGinley, David, & McCormick, 2015; Reimer et al., 2016; Vinck, Batista‐Brito, Knoblich, & Cardin, 2015) have revealed correlations between pupil diameter and the activity of subcortical neuromodulatory nuclei, including the noradrenergic locus coeruleus. These neuromodulatory systems project extensively to the rest of the brain and exert powerful effects on the responsivity (or gain) of neural processing (Aston‐Jones & Cohen, 2005b; Mather, Clewett, Sakaki, & Harley, 2016; Servan‐Schreiber, Printz, & Cohen, 1990). Moreover, theoretical studies have shown that gain modulation is a candidate neural mechanism for regulating the trade‐off between the speed and accuracy of behavior: a state of high neural gain, which is produced by strong neuromodulatory activity and associated by proxy with relative dilation of the pupil, can produce fast but inaccurate behavior; low gain states, on the other hand, are associated with slower but more accurate behavior (Eckhoff, Wong‐Lin, & Holmes, 2009; Niyogi & Wong‐Lin, 2013; Standage, You, Wang, & Dorris, 2011). Indeed, results from previous neuroimaging and single‐unit experimental studies are broadly consistent with gain modulation as a plausible neural mechanism for implementing adjustments in the speed‐accuracy trade‐off (Forstmann et al., 2008; Heitz & Schall, 2012; Thura & Cisek, 2016).

These causal and indirect accounts of a potential correlation between pupil size and the speed and accuracy of behavior are not mutually exclusive and may work in tandem. In any case, we have recently provided converging evidence for the existence of such a correlation. In one study (Murphy, Boonstra, et al., 2016), participants made difficult perceptual decisions that required protracted deliberation about the direction of motion of a noisy cloud of moving dots and were incentivized to be either fast or accurate in doing so. Both tonic (prestimulus, baseline) pupil size and phasic (poststimulus, decision‐related) pupil dilation were highly sensitive to the incentivization scheme, with heightened speed emphasis associated with larger pupils in both cases. In another study (Naber, Hommel, & Colzato, 2015), participants performed a visuo‐motor aiming task requiring rapid movement of a mouse cursor to a clearly marked target location on a screen. Unlike the motion discrimination task, this task does not require a gradual decision process in the face of sensory uncertainty but does necessitate more complex and protracted motor behavior. Critically, participants in this study were also administered choline supplements to boost cholinergic activation. Relative to placebo, this manipulation was associated with both decreased tonic pupil size (likely at least in part through activation of the cholinergic pupillary sphincter muscle) and slower but more accurate responses. Moreover, the choline effect on pupil size correlated with the choline effect on behavior across subjects. Thus, pupil size in both studies was associated with adjustments in the trade‐off between the speed and accuracy of behavior, despite the task contexts differing considerably.

In the present work, we sought to build on these initial findings by incorporating key elements from both studies described above. Specifically, a new cohort of participants performed the same visuo‐motor aiming task used in Naber et al. (2015) but now under different levels of speed emphasis that were encouraged via a combination of task instruction and varying incentive schemes. In this respect, the present study is similar to Murphy, Moort, et al. (2016), but it also extends that work in two ways. First, we implemented an extensive single‐trial analysis to link both baseline pupil size and trial‐evoked pupil dilation responses to both external (i.e., incentives) and internal (i.e., trial‐by‐trial fluctuations within each task condition) changes in the speed‐accuracy trade‐off. Second, the visuo‐motor task, which requires more complex than standard button presses in reaction time tasks, allows a novel analysis in which the participants’ responses are decomposed in multiple motoric phases and separately linked to the timing of distinct pupil response profiles. We predicted that both tonic and phasic pupil size would increase with the degree of speed pressure and, moreover, that fluctuations in pupil size within each level of speed emphasis would correlate with the speed and accuracy of visuo‐motor responses. Such observations would provide additional evidence for a general role of pupillary systems in regulating the speed‐accuracy trade‐off across varied behavioral contexts.

## 2 METHOD

### 2.1 Participants

Twenty human individuals were invited to participate in the experiment. Three participants were excluded from analysis because they misunderstood the instructions during the experiment. The remaining 17 participants were all right‐handed, young students (age *M* = 19.94, *SD* = 1.64; 10 female) and had normal or corrected‐to‐normal vision. Participants were naïve to the purpose of the experiment, gave informed written consent before participation, and received either study credit or money ($10) after participation. The experiments conformed to the ethical principles of the Declaration of Helsinki and were approved by the local ethical committee of Harvard University.

### 2.2 Apparatus and material

Stimuli were generated on a 21‐inch Samsung Syncmaster CRT screen (Samsung, Seoul, South Korea) with a Dell computer (Dell, Round Rock, TX) operating Windows 7 (Microsoft, Redmond, WA) and MATLAB (MathWorks, Natick, MA). The presentation monitor displayed 1,600 × 1,200 pixels at a 60 Hz refresh rate. Screen size was 44 cm in width and 33 cm in height (35 × 26.5 visual degrees), and the participant's viewing distance to the screen was fixed with a chin and forehead rest at 70 cm. The screen cursor movements were controlled with a standard Dell computer mouse with an optical sensor and USB cable at approximately 5500 Hz. Pupil size and gaze position of one eye was monitored with an EyeLink 1000 desktop mounted eye tracker (SR Research, Osgoode, ON, Canada) at a rate of 1000 Hz.

### 2.3 Stimuli and procedure

**Figure 1:**
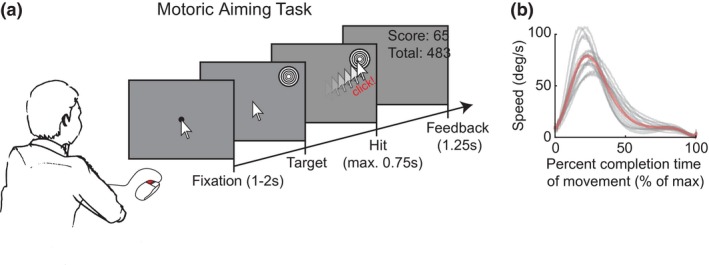
Stimuli, procedure, and movement characteristics. (a) Participants viewed a computer screen and used an optical mouse to quickly and accurately move a cursor from fixation to a target's center on the screen. Participants received points for fast response times and accurate (near‐)target hits. Movements were characterized by two phases: a fast ballistic movement followed by a slower error‐correction movement. (b) Speed profiles of a movement as a function of the percentage completion of movement. A movement started when the mouse velocity crossed a threshold (0%) and ended when participants clicked on the target (100%). Gray lines display the average speed across trials (i.e., across all possible target locations) per participant, and the red line displays the group average

We tested participants on a motoric coordination task that took approximately 45 min to complete. The task consisted of a visuo‐motor aiming task, very similar to CANTAB’s motor screening task (i.e., an assessment part of the Cambridge neuropsychological test automated battery; Cambridge Cognition Ltd., Cambridge, UK). Participants used their right hand to move a digital, computer mouse‐controlled screen cursor (pointer) to a target as fast and close to its center as possible. Once arrived at the target, participants had to click the mouse to finalize the trial and gain points (Figure 1a). A trial started with the presentation of a blank gray screen, a fixation dot (10 pixels wide, ~0.2 visual degrees), and the mouse cursor for a random duration of 1–2 s. Participants were instructed to maintain fixation until the target was shown. The mouse cursor could not be moved away from fixation until the target was shown. The fixation dot disappeared when the target was shown. The target was equiluminant to its background and consisted of a 100‐pixel wide bull’s‐eye (~2.2 visual degrees) with rings that alternated in black and white as a function of eccentricity. The target was shown at a random screen position (i.e., at any location between the fixation dot and screen border), and its appearance released the mouse cursor such that participants could move the cursor to the target. The goal was to click the left mouse button after the cursor was brought to the target, as fast and as close to the target's center as possible with an emphasis that varied depending on task instruction (see below).

Participants had to increase a total score with points that could be collected by producing a fast and accurate movement per trial. They received points from a range of 0 to 50 per trial depending on their response time (linear relationship; e.g., 250 ms = 50 points, 500 ms = 25 points, 750 ms = 0 points; 1 point per 10 ms). A trial was automatically aborted and a penalty of −100 points was given when a participant did not hit the target within 750 ms. Participants additionally received points from a range of 0 to 50 per trial depending on their accuracy (also a linear relationship; e.g., trials with target hits at a distance of 50 pixels from its center got 0 points, trials with 0 pixels distance got 50 points; 1 point per pixel). A penalty of −100 points was given when participants clicked the cursor outside the target (>50 pixels). To keep participants motivated, they received visual feedback for 1.25 s after their response about the points received in the current trial and their total score accumulated in previous trials. The task consisted of a total of 600 trials, and each trial automatically started after the feedback. The experiment was divided in six blocks that each consisted of 100 trials. Depending on the block, participants received three different instructions: be fast, be accurate, or trade off both speed and accuracy. To stimulate the participants to adhere to these instructions, the given points per trial were separately weighted for speed and accuracy depending on the instruction. The speed score was multiplied with a factor 1 and the accuracy score with a factor 0.5 in the fast instruction condition. A factor of 0.5 for speed and 1 for accuracy scores was used in the accurate instruction condition, and a factor 0.75 was used for both speed and accuracy scores in the trade‐off instruction condition. The factors 0.5, 0.75, and 1 and not 0, 0.5, and 1 were chosen to ensure that participants always traded off speed and accuracy, though at different criterion thresholds, in all trials. The instruction conditions were assigned randomly to each block, with a total of two blocks per condition per subject. Preceding the experiment, participants received time to practice some trade‐off trials until they were confident that they understood the task.

### 2.4 Analysis

Several dependent variables were extracted from the data. Response time (RT), accuracy, move distance, move speed, and peak velocity of the movement were analyzed. RT was based on the median time between target onset and target hit across all correct trials per participant. We chose to initially focus on RT (as opposed to RTs and movement times separately) because this measure matches best with those reported in previous studies investigating the relationship between pupil‐linked arousal and behavior (e.g., Murphy, Boonstra, et al., 2016). A trial was correct when the click was both within 50 pixels of the target's center and faster than 750 ms. Accuracy was based on the Euclidian distance in percentage (0% = 50 pixels, 100% = 0 pixels) between the cursor's position and target's center at the time of the mouse click. Move distance was operationalized as the Euclidian distance between the cursor's position at movement onset and the cursor's end position. The moment of movement onset was chosen as the time at which the velocity of the cursor crossed a trial‐dependent velocity threshold (>30th percentile of all velocity values within a trial). Trials in which movement onsets were faster than 100 ms were labeled as false starts (i.e., faster than the visual system can process the target's location) and were excluded from the analysis (average trials removed: *M* = 0.16%, *SD* = 0.27%). Peak velocity was the maximum velocity value during the movement per trial. Velocity values were based on the difference in Euclidian distance between consecutive cursor positions divided by the difference in time (~1/5,500 s). Velocity values were smoothed within a moving average convolution window of 200 time points. The reaction time (onset time of movement) and duration of the movements (which sum to yield the total RT for a trial) were also inspected separately.

As shown in Figure 1b, movement trajectories through space typically show two phases: a ballistic and an error‐correction movement (Woodworth, 1899). The onset of the error‐correction phase on each trial was detected by computing the timing of the minimum of the trough in the bimodal velocity profile. Note that the velocity trough is less apparent in Figure 1b because there it is smoothed out via across‐trial averaging, but it was clear and easy to identify for most trials (error‐correction movement detected on average across participants: *M* = 86%, *SD* = 7%).

Pupil diameter was measured in arbitrary units by the eye tracker. Missing data during periods of blinks were interpolated using a cubic spline fit. The effect of gaze angle on pupil size (i.e., pupils with rotated eye away from the camera appear smaller than pupils staring directly at the camera) was removed from the pupil data by regressing out the effects using *X*‐ and *Y*‐gaze coordinates measured during calibration. Tonic, baseline pupil size per trial was calculated as the average pupil size in the time window 500 ms before target onset. Phasic pupil size was analyzed from −0.5 to 3 s relative to target onset. To enable comparisons of phasic pupil responses within and between participants, pupil size was baseline corrected by subtracting pupil size at target onset from the full pupil trace per trial (i.e., from −0.5 s to +3.0 s around target onset).

Differences between dependent variables across instruction conditions were assessed with a one‐way repeated measures analysis of variance (ANOVA) and post hoc *t* tests. Correlations were computed with the Pearson correlation coefficient and transformed to Fisher’s *z* values when multiple correlations were compared to zero. Regression coefficients representing the relationships between task behavior and either average tonic pupil size or phasic pupil size per trial were estimated per subject in general linear models (GLM). The coefficients (i.e., betas) for relationships of phasic pupil size were calculated as a function of time around stimulus onset. Depending on the purpose of the GLM analysis, the predictors could consist of response time, accuracy, a speed‐accuracy trade‐off (SAT) measure, a movement performance measure, and/or target eccentricity (i.e., the Euclidian distance between the centers of fixation and target in visual degrees) per trial. The SAT measure was calculated as normalized speed (a score of 0 for the slowest trial, 1 for the fastest trial) minus the normalized accuracy per subject (a score of −1 for the most accurate trial, 0 for the least accurate trial). A SAT score of 1 indicated that the SAT was biased toward speed and −1 indicated a bias toward accuracy. The movement performance measure was calculated as normalized speed plus normalized accuracy, with a score of 2 for trials with the fastest and most accurate movement and 0 for slowest and least accurate performance. Target eccentricity was included in each GLM to absorb shared variance in pupil size and behavior that was driven by this experimental factor. To compare the latencies at which the pupil exhibited sensitivity to ballistic and error correction movements, the timing of betas exceeding a fixed threshold of 50% of the maximum average betas was calculated per participant.

## 3 RESULTS

### 3.1 Movement trajectories

**Figure 2:**
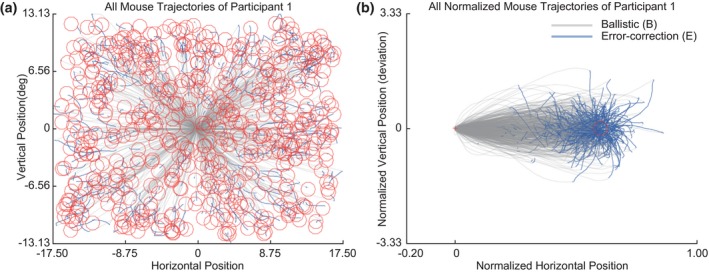
Mouse trajectories and movement characteristics of an exemplar participant. A cursor was moved from screen center (fixation) toward targets (red circles) that could appear at any location across the screen. Here are shown all movements of a typical participant. (b) Resulting trajectories of the movements can be divided in a ballistic (gray) and error‐correction (blue) submovement. Differences in movement characteristics between these phases become evident after transforming space (rotation and normalization) such that all orientations and distances toward the target locations are aligned and the same

Participants rapidly and accurately moved a digital mouse cursor to a total of 600 targets that appeared one by one at a random location on a visual display screen. Such coordinated movements to visual stimuli typically contain several characteristics, including an initial ballistic and a subsequent error‐correction phase (Woodworth, 1899). Here, we first explored whether such and several other patterns were evident in the mouse movements across all participants. Figure 2a shows the trajectories of the mouse movements divided in ballistic (gray) and error‐correction (blue) submovements from the fixation cross to the targets (red). Each submovement showed a distinct pattern of movement characteristics. The ballistic movement occurred predominantly in the first 197 ms (averaged across trials per participant and then averaged across participants; *SD* = 13 ms), which is 60% (*SD* = 7%) of the total movement time. In this phase, 9.7 degrees (*SD* = 0.6°) is covered in distance (though this varies with distance of the target from fixation), which corresponds to 92% (*SD* = 2%) of the total distance moved. To demonstrate the spatial dynamics of these movements, Figure 2b shows the movement trajectories of all trials of Participant 1 (all other participants showed very similar behavioral patterns). As demonstrated in this figure, the majority of ballistic movements were in the direction of the target. Some of the ballistic movements initialized in a more deviant direction and had highly curved trajectories that not always reached the target in time. Figure 2b also highlights second error‐correction movements (blue) that occur after the initial ballistic movements. These movements lasted 132 ms (*SD* = 32 ms) and covered 0.8 degrees (*SD* = 0.3°) of distance on average. The error‐correction movements are marked by relatively more curved trajectories than the ballistic movements. In sum, ballistic movements are fast, distant, and relatively inaccurate, while error‐correction movements are slow, short, and accurate.

### 3.2 Effects of speed‐accuracy instructions on response times, accuracy, and movement properties

We next assessed the effects of task instructions on each submovement. Depending on the block, participants were instructed to be either fast, accurate, or to balance and trade off the speed and accuracy of their movements, and participants may have also fluctuated in this balance across trials within a block. In the following, we investigate whether the participants’ behavior altered according to the speed‐accuracy trade‐off.

#### 3.2.1 Behavioral performance

**Figure 3:**
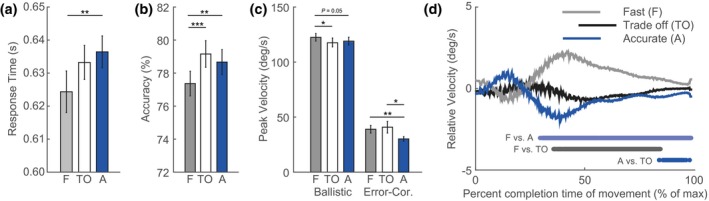
Behavioral performance measures per speed/accuracy instruction. Means and standard errors across participants are shown for (a) response times, (b) accuracy, and (c) movement peak velocity per instruction to be either fast (F; gray) or accurate (A; blue), or to trade off both speed and accuracy (TO; white). (d) Average relative velocity (subtracted average) of movements per instruction condition is plotted over time. Patches at the bottom of the panel indicate at which time points the velocity traces differed significantly between conditions (pair‐wise comparison, *p < .*05)

On average, 86% of the targets were hit in time (<750 ms) and within the target border (<2.2°). Accuracy and response times weakly but significantly correlated across trials per participant (average correlation across participants: 0.07 ± 0.10; *t*(16) = 2.82, *p* = .012) meaning that slow trials tended to be accurate and vice versa. Instruction also significantly affected response times (repeated measures ANOVA: *F*(2, 32) = 4.48, *p* = .019; and accuracy, *F*(2, 32) = 15.09, *p* < .001). When participants were instructed to be fast, response times decreased as compared to when they were instructed to be more accurate (Figure 3a; two‐sided paired *t* test: *t*(16) = 3.19, *p = .*006; for means and standard deviations, see Table S1 in online supporting information). Participants were also less accurate when instructed to be fast as compared to accurate (Figure 3b), *t*(16) = 3.34, *p = .*004, and trade‐off conditions, *t*(16) = 5.00, *p < .*001. The response times and accuracy scores did not differ between the trade‐off and accuracy instruction condition (*p* > .05), probably due to the fact that there is large variance across participants in how they set their criterion with respect to the accuracy instruction condition (some participants expressed a bias for accuracy). Nonetheless, the fast and accurate instruction had the expected effects on response times and accuracy.

#### 3.2.2 Decomposition of response time

Response time as analyzed above is a composite measure that reflects the combination of discrete components of the visuo‐motor process governing responses on our task, including movement initiation, the duration of which is captured by movement onset time (the reaction time), the initial ballistic movement, and the final error correction movement. We next examined which of these components varies across instruction conditions. We found no effects of instruction on reaction time (i.e., duration until movement onset; *F*(2, 32) = 0.84, *p = .*438) and peak velocity collapsed across ballistic and error correction movements, *F*(2, 32) = 1.09, *p = .*349, but a significant effect on movement duration that went in the same direction as the effect on total response times, *F*(2, 32) = 5.39, *p = .*010; *M*_fast_ = 0.351, *M*_TO_ = 0.360, *M*_acc _= 0.364; fast versus accuracy (acc): *t*(16) = 3.75, *p = .*002; fast versus trade‐off (TO): *t*(16) = 1.88, *p = .*08; TO versus acc: *t*(16) = 1.03, *p = .*320. When decomposing movements even further, we observed a significant difference across instruction conditions only for average peak movement velocity of the ballistic, *F*(2, 32) = 3.38, *p = .*037, and error‐correction movements, *F*(2, 32) = 4.61, *p = .*018. The fact that we found an effect of peak velocity for the individual components but not when components were collapsed may seem surprising. However, the individual peak velocities of the ballistic and error‐correction components do not correlate across trials (average correlation across participant: *M* = 0.05, *SD* = 0.22; *t*(16) = 0.85, *p = .*409). Inspection and post hoc condition comparisons of the peak velocity of ballistic submovements indicated a trend toward higher peak velocities in the fast condition as compared to the accurate, *t*(16) = 1.71, *p = .*053, and trade‐off conditions, *t*(16) = 2.38, *p = .*015 (Figure 3c). Peak velocity of error‐correction movements was decreased in the accurate instruction condition as compared to the fast, *t*(16) = 3.26, *p = .*002, and trade‐off conditions, *t*(16) = 2.47, *p = .*013. These results suggest that instructions mostly affected the duration of movements, and that, with respect to the trade‐off condition, peak velocity of a ballistic submovement increased when participants were instructed to be fast and that the peak velocity of an error‐correction submovement decreased when instructed to be accurate. The relative velocity across instruction conditions as a function of time from movement onset, as plotted in Figure 3d, further confirms the pattern that speed instructions affect early/intermediate phases of a movement while accuracy instructions affect later phases of a movement.

### 3.3 Pupil dynamics and the speed‐accuracy trade‐off

#### 3.3.1 Tonic, baseline pupil size

**Figure 4:**
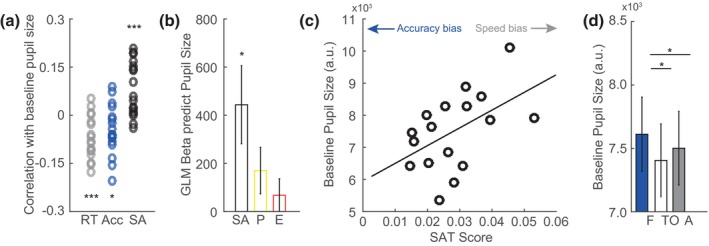
Effects of speed and accuracy on tonic, baseline pupil size. (a) Baseline pupil size correlated with response times (RT), accuracy (Acc), and speed‐accuracy trade‐off (SA) across trials per participant (circles). (b) GLM betas, averaged across participants, indicated significant explained variance in the single‐trial pupil response by speed‐accuracy trade‐off (SA) but not by performance (P) and target eccentricity (E). (c) Average SAT scores correlated with average baseline pupil size across participants (circles). (d) Baseline pupil size was largest when participants were instructed to be fast

Our next analysis scrutinized the relationship between the speed‐accuracy trade‐off and pupillary dynamics. First, we looked at whether baseline, tonic pupil size predicted the speed and accuracy of movements. We computed correlations between tonic pupil size and accuracy and response times across all trials per participant (Figure 4a). The average correlation coefficients across participants were in the expected directions (RT: −0.07 ± 0.07; accuracy: −0.05 ± 0.08; SAT scores: 0.08 ± 0.08) and differed significantly from zero (RT: *t*(16) = 4.30, *p < .*001; accuracy: *t*(16) = 2.44, *p = .*027; SAT: *t*(16) = 4.01, *p = .*001). Collectively, this indicates that participants made faster but less accurate movements when having relatively large baseline pupil size (for average accuracies, RTs and SAT scores across participants per level of pupil size, see supporting information Figure S1). To further investigate whether baseline pupil size predicted the speed‐accuracy trade‐off of movements, independent of target eccentricity and movement performance, we calculated the betas from a general linear regression model, investigating the relationship of tonic pupil size with SAT, performance, and target eccentricity across trials per participant (Figure 4b). Baseline pupil size was significantly predicted by SAT but not by performance and target eccentricity (average beta for SAT: 444 ± 670, *t*(16) = 2.73, *p = .*015; performance: 170 ± 400, *t*(16) = 1.76, *p = .*098; target eccentricity: 68 ± 281, *t*(16) = 1.00, *p = .*334). SAT scores also correlated significantly with baseline pupil size across participants (Figure 4c, *r*(15) = 0.52, *p = .*034), indicating that participants with relatively large pupils have a bias toward fast and less accurate movements than participants with smaller pupils. Baseline pupil size was also larger when participants were given the instruction to be fast as compared to other instructions (Figure 4d; *F*(2, 32) = 5.84, *p = .*007; fast versus accurate: *t*(16) = 2.40, *p = .*029; fast versus trade‐off: *t*(16) = 2.64, *p = .*018). To summarize the results above, a relatively large pupil before target onset, in relation to other trials or other individuals, leads to faster response times but less accurate movements.

#### 3.3.2 Phasic pupil responses

**Figure 5:**
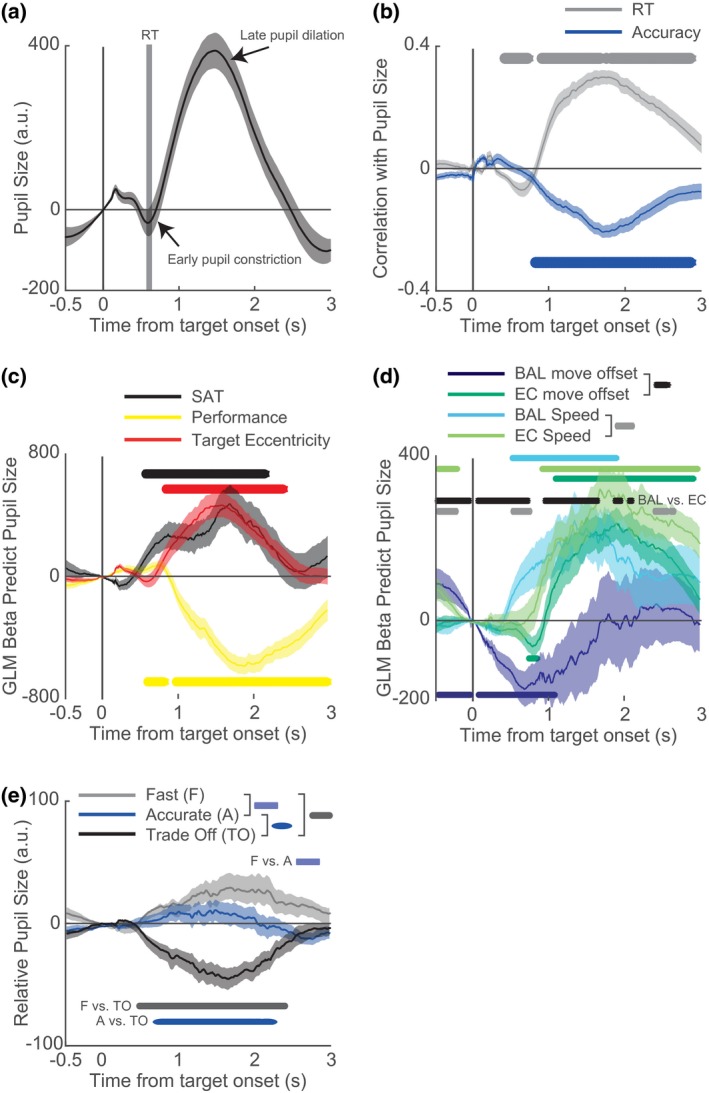
Effects of speed and accuracy on phasic pupil responses. (a) Average of baseline‐corrected pupil size across participants (pupil size was first averaged across trials per participant) changed in response to the target by constricting, followed by a strong pupil dilation response after the target was hit. (b) Time‐resolved correlations (Pearson's *r*) between pupil response and both RT (gray) and accuracy (blue), averaged across participants, indicated significant explained variance in the single‐trial pupil response by these features of behavior. (c) GLM betas also indicated explained variance by several performance components. (d) The same applied to several movement properties, including the timing of offsets and speed of ballistic (BAL) and error‐correction (EC) components. (e) Average relative pupil size (i.e., average pupil size across all conditions in the 500‐ms time window before stimulus onset was subtracted) changed as a function of the speed‐accuracy instructions to be fast (gray), accurate (blue), or to trade off between speed and accuracy (black). (a‒e) Relative pupil size (i.e., baseline‐corrected pupil size per instruction condition minus average of baseline‐corrected pupil across all conditions) is here depicted to better visualize the differences between instruction conditions. Half‐transparent patches around the average traces indicate standard errors from the mean. Bars below and above the traces indicate at which time points the traces differed significantly from zero (b‒d) or between conditions (d‒e) (*p < .*05), respectively

We next examined whether trial‐evoked changes in pupil size could also be linked to the speed and accuracy of movements. Pupil size changed in two phases (Figure 5a): first, an early small pupil constriction in response to stimulus onset (e.g., see Naber & Nakayama, 2013) that peaked at approximately 600 ms following target onset, followed by a late strong pupil dilation (e.g., Murphy, Boonstra, et al., 2016). To investigate when the trial‐evoked change in pupil size was related to the speed and accuracy of motor aiming, we calculated correlations as a function of time from target onset (Figure 5b). Note that the impulse response of pupil dilation to an eliciting event is relatively slow, peaking after approximately 1 s and gradually decaying thereafter (de Gee, Knapen, & Donner, 2014; Hoeks & Levelt, 1993; Murphy, Boonstra, et al., 2016; Wierda et al., 2012). Thus, while all analyzed motor responses on our task were completed within 0.75 s of target onset, it is expected that variance in the phasic pupil response that is driven by cognitive processes culminating in a motor aiming response will be present for a prolonged period after response execution.

Within the period of 0.5 to 1.0 s after target onset, the correlation patterns with phasic pupil responses were comparable to those seen earlier for tonic pupil size, that is, a negative relationship with accuracy and response times. However, after 1 s, the relationship with response times flips positive while the relationship with accuracy remains negative. To confirm that the pattern of relationships before 1 s reflects a speed‐accuracy trade‐off while taking into account other covariables, we created a GLM predicting pupil size per time point with the normalized predictors of SAT score, movement performance, and target eccentricity. Figure 5c shows the average betas across participants as a function of time around target onset per condition. First, we found that SAT was positively related to pupil size, showing a biphasic distribution of coefficients as a function of time. This means that pupil size increases with a peak around 1 s and increases even more with a peak around 2 s when the trade‐off is biased toward speed. Note that the dilatory impulse response of the pupil typically peaks approximately 1 s after the eliciting event (Hoeks & Levelt, 1993). It is thus likely that the early reflection of a speed bias in the pupil, which manifests from ~0.75 to ~1.25 s, relates mostly to the initial ballistic movement (typically occurring within the first 200 ms after target onset), while the late correlation with SAT around ~1.50 to ~2.50 s relates to the later error‐correction movement. This dissociation is supported by results from an additional GLM plotted in Figure 5d, showing that the speed of initial ballistic movements correlates with pupil size at earlier time points (50% beta threshold of ballistic speed was exceeded on average at *M* = 0.77 s, *SD* = 0.33) than the speed (*M* = 1.13 s, *SD* = 0.40; *t*(16) = 2.27, *p = .*049) and of subsequent error‐correction movements. A similar discrepancy was observed for the beta patterns of ballistic versus error‐correction move offsets, but the immediate effect on ballistic move offset (i.e., likely an effect of pupil baseline; see blue significance bars at the bottom of Figure 5d) hampers the accurate detection of when betas exceeded thresholds.

We also found that movement performance was positively though marginally correlated with the phasic pupil response before 1 s after stimulus onset, and this correlation flips in direction after approximately 1 s, with a peak pupil size increase after 2 s when performance was low. The late phasic pupil response was also positively associated with error‐correction offset latency. This latter association may indicate postmovement monitoring processes, which we further expand on in the Discussion section.

As a last analysis, we examined the effect of speed‐accuracy instruction on phasic pupil responses. The late pupil dilation response was significantly modulated by instructions (Figure 5e). Pupils dilated more when participants were instructed to be fast as compared to accurate; while this effect manifests numerically from approximately 600 ms following target onset and is nicely in line with the SAT account of pupil dynamics, we note that it reaches statistical significance only very late in the analyzed epoch, from around 2.5–3.0 s after target onset. Moreover, we observed that the trade‐off instruction resulted in the weakest relative pupil dilation across the three experimental conditions, which is not immediately consistent with the SAT account. This complex pattern of task instruction effects may be at least partly due to the ways in which condition‐related differences in average response latencies and/or expected rewards manifest in the stimulus‐aligned pupil signal. Rather than speculate about this result further, we leave the investigation of this effect to future work.

To summarize the results above, phasic pupil responses were related to the trade‐off between the speed and accuracy of motor aiming over a sustained period following target onset, which likely reflects combined effects on both initial ballistic and subsequent error‐correction movements. We also observed a strong negative relationship between the later parts of the pupil response and task performance, which may reflect a mixture of effects related to urgency and performance monitoring, that we elaborate on below.

## 4 DISCUSSION

We have found that tonic pupil size and phasic pupil responses track fluctuations in the speed and accuracy trade‐off across trials in a visuo‐motor aiming task. Explicit manipulations of this trade‐off via changes in the payoff structure of the task also affect tonic pupil size and phasic pupil responses. Specifically, increases (or decreases) in baseline pupil size before target onset or subsequent transient increases (or decreases) in pupil size after visual targets are linked to faster (or slower) and less (or more) accurate responses. This supports our hypothesis that a change in the speed‐accuracy trade‐off will be reflected in both tonic and phasic changes in pupil size.

Our visuo‐motor task has three appealing aspects: (a) both speed and accuracy can be probed per trial, (b) both the speed‐accuracy trade‐off (through instruction and natural fluctuations) and stimulus processing (through stimulus location) can be manipulated, and (c) the biphasic motor response provides insights into the time course of the effects of speed and accuracy on pupil size. These benefits enabled us to reveal several novel relationships.

First, we show that tonic, baseline pupil size depends on the speed‐accuracy trade‐off set by the participant per trial. These effects are in line with previous findings by studies with humans (Murphy, Boonstra, et al., 2016; Naber et al., 2015) and rodents (Schriver, Bagdasarov, & Wang, 2018) and suggest that the pupil's sensitivity adjustments in the speed‐accuracy trade‐off is not limited to a specific task context. Aside from this finding, several other aspects of our results warrant further consideration. We have found that response times are faster when baseline pupil size is large. However, previous reports have shown both the opposite pattern (Gilzenrat et al., 2010) and nonlinear relationships between RT and baseline pupil size (Murphy, Robertson, Balsters, & O'Connell, 2011; van den Brink, Murphy, & Nieuwenhuis, 2016). This inconsistency can perhaps be explained by differences in stimuli and task design and to what degree these evoked variations in arousal. One low‐level difference compared to previous work is that we presented visual targets for which it is known that higher intensity (i.e., increased luminance, both externally through experimenter control but also potentially internally due to increased pupil size) scales with faster response times (Vaughan et al., 1966). By contrast, some previous studies used auditory stimuli, the perceived intensity of which may not be directly affected by pupil size. Hence, the size of the pupil could in our case have directly altered perception, which subsequently affected response times. Another possible explanation for the discrepancy with previous work is that previous studies have tended to use sustained attention tasks that were designed to elicit strong fluctuations in alertness and arousal. It is possible that the current task sampled only a limited portion of the arousal continuum (Yerkes & Dodson, 1908) wherein subtle fluctuations in arousal state have a monotonic effect on behavior.

Turning to the evoked pupil responses on the motor aiming task, these showed a biphasic pattern with an early constriction likely due to luminance contrast transients introduced by the stimulus and a later, sustained dilation likely driven by stimulus processing and motor execution. We were able to show through single‐trial analyses that, over and above this trial‐averaged response, variation in poststimulus pupil change was strongly related to the trade‐off between the speed and accuracy of motor aiming, again supporting our initial hypothesis. The variation in strength of the link between pupil size at different latencies relative to target onset and different components of the motor aiming action suggest that the pupil displays a delayed response (Hoeks & Levelt, 1993) to two time‐separated processes: early dilation (or lack of constriction) is likely associated with faster and lower‐latency ballistic movements, while later dilation is likely associated with faster but late error‐correction movements. Thus, the observed trial‐related pupil/arousal response may in fact consist of discrete temporal components, each of which is modulated by sensory‐motor operations that unfold in rapid succession. Although out of the scope of the current study, the creation of a deconvolution model that simulates pupil dilation patterns as convoluted responses to discrete trial events could shed further light on the temporal dynamics of these associations (e.g., Wierda et al., 2012).

We also observed that the later phase of the dilation response was strongly negatively related to motor aiming performance on our task. This association, together with the positive relationship between this pupil response and error‐correction latency, is likely related to a combination of different factors. First, again consistent with a possible role for phasic pupil‐linked arousal in rapidly modulating the speed‐accuracy trade‐off, we have recently shown during two‐alternative decision making under a deadline that pupil dilation is especially large when the decision process is taking a long time, and this is linked to the generation of urgency that ensures timely response execution at the expense of diminished choice accuracy (Murphy, Moort et al., 2016). Such a building urgency signal may also have been invoked by our participants to meet the stringent deadline imposed by the motor aiming task (750 ms), would be particularly strong during error correction movements, and may contribute to the association that we observed between late pupil dilation and both slower move offsets (where greater urgency is generated to successfully meet the deadline) and poorer aiming accuracy (which is a consequence of that urgency).

Second, it is likely that the late pupil dilation is strongly driven by processing of feedback about the speed and accuracy of the motor response, which we presented immediately upon response execution. It is well known that the pupil is highly sensitive to negative events and feedback, prediction errors, and surprise (Derksen, van Alphen, Schaap, Mathot, & Naber, 2018; Kloosterman et al., 2015; Koenig, Uengoer, & Lachnit, 2018; Lavín, San Martín, & Rosales Jubal, 2014; Murphy, van Moort, et al., 2016; Nassar et al., 2012; Preuschoff, t Hart, & Einhäuser, 2011; Wessel, Danielmeier, & Ullsperger, 2011), and accordingly we observed that late pupil dilations tended to be largest when the performance on a given trial was lowest. This error‐related pupil response has elsewhere been linked to next‐trial behavioral adjustments on a choice RT task (Murphy, van Moort, et al., 2016). We speculate it may play a similar role during motor aiming and plan to address this question in future work.

A third factor that we acknowledge may be responsible for the sensitivity of late pupil dilation to RT relates to the relatively slow nature of the dilation impulse response (Hoeks & Levelt, 1993). In the special case that the input to the pupil system has a constant amplitude but a duration that scales with RT (which is consistent with the form of the input identified in some decision‐making tasks; de Gee et al., 2014; Murphy, Boonstra, et al., 2016), a spurious positive correlation between pupil dilation and RT would be observed.

In sum, there are several candidate explanations for the observed pattern of late pupil response—relating to urgency, feedback processing, and the pupil impulse response function—that are not mutually exclusive. Irrespective of the specific combination of factors driving the late pupil response, however, an important implication of our results is that the trial‐related phasic arousal response likely modulates multiple stages of motor aiming that play out in rapid temporal succession, resulting in multifaceted effects on observed behavior.

Cognitive effects on dilatory pupil responses are often interpreted in terms of noradrenergic, locus coeruleus activity (e.g., Einhäuser et al., 2008; Laeng, Sirous, & Gredebäck, 2012; Murphy, Robertson, Balsters, & O'Connell, 2011). A common perspective is that activity of the locus coeruleus, the primary source of cortical noradrenaline, not only exerts a powerful modulatory influence on brain state (Aston‐Jones & Cohen, 2005b; McGinley et al., 2015) but also triggers the sympathetic nervous system to dilate the pupil through activation of the dilator muscle in the iris (Gilzenrat et al., 2010; Jepma & Nieuwenhuis, 2011). Indeed, the nature of the associations between both baseline and trial‐related pupil responses and aspects of the speed‐accuracy trade‐off that we report here and elsewhere (Murphy, Moort, et al., 2016) are consistent with the modulatory effect that noradrenaline has been proposed to have on the gain of neural processing (Aston‐Jones & Cohen, 2005a; Eckhoff et al., 2009; Servan‐Schreiber et al., 1990; Standage et al., 2011). While considerable experimental evidence linking locus coeruleus activity and pupil size has been reported (de Gee et al., 2017; Joshi et al., 2016; Murphy, O'Connell, O'Sullivan, Robertson, & Balsters, 2014; Rajkowski, Kubiak, & Aston‐Jones, 1993; Reimer et al., 2016; Varazzani et al., 2015), however, it is also possible that the cholinergic system exerts control over pupil size in visuo‐motor tasks (McGinley et al., 2015; Naber, Frässle, et al., 2013; Naber et al., 2015; Nelson & Mooney, 2016; Nieuwenhuis, de Geus, & Aston‐Jones, 2011; Reimer et al., 2016; Vinck et al., 2015). Changes in pupil size in response to task difficulty or explorative behavior in visual and auditory paradigms have previously been attributed to processes in the cholinergic, parasympathetic pathway including the Edinger‐Westphal nucleus rather than the adrenergic, sympathetic pathway (Steinhauer, Siegle, Condray, & Pless, 2004; Wang et al., 2016). Importantly, we also recently demonstrated that administration of choline supplementation, which likely boosts systemic cholinergic availability, both decreases resting pupil size and changes behavior toward a greater emphasis on accuracy rather than speed on precisely the same motor aiming task used presently (Naber et al., 2015). As such, we deem it most appropriate to leave open the question of which neuromodulatory systems might drive the pupillometric effects reported here.

## Supporting information


**Figure S1 **Behavioral performance per binned percentile pupil size. Means and standard errors are shown.

**Table S1 **Behavioral performance per speed/accuracy instruction. Means and standard deviations are shown per behavioral performance measures and per instruction.

## References

[psyp13499-bib-0001] Aston‐Jones, G. , & Cohen, J. D. (2005a). Adaptive gain and the role of the locus coeruleus‐norepinephrine system in optimal performance. Journal of Comparative Neurology, 493(1), 99–110. 10.1002/cne.20723 16254995

[psyp13499-bib-0002] Aston‐Jones, G. , & Cohen, J. D. (2005b). An integrative theory of locus coeruleus‐norepinephrine function: Adaptive gain and optimal performance. Annual Review of Neuroscience, 28, 403–450. 10.1146/annurev.neuro.28.061604.135709 16022602

[psyp13499-bib-0003] Beatty, J. (1982). Task‐evoked pupillary responses, processing load, and the structure of processing resources. Psychological Bulletin, 91(2), 276 10.1037/0033-2909.91.2.276 7071262

[psyp13499-bib-0004] Beatty, J. , & Lucero‐Wagoner, B. (2000). The pupillary system In Cacioppo J. T., Berntson G., & Tassinar L. G. (Eds.), Handbook of psychophysiology (2nd ed., pp. 142–162). Hillsdale, NJ: Cambridge University Press.

[psyp13499-bib-0005] Binda, P. , Pereverzeva, M. , & Murray, S. O. (2013). Attention to bright surfaces enhances the pupillary light reflex. Journal of Neuroscience, 33(5), 2199–2204. 10.1523/jneurosci.3440-12.2013 23365255 PMC6619119

[psyp13499-bib-0006] Breton‐Provencher, V. , & Sur, M. (2019). Active control of arousal by a locus coeruleus GABAergic circuit. Nature Neuroscience, 22(2), 218 10.1038/s41593-018-0305-z 30643295 PMC6385895

[psyp13499-bib-0008] de Gee, J. W. , Colizoli, O. , Kloosterman, N. A. , Knapen, T. , Nieuwenhuis, S. , & Donner, T. H. (2017). Dynamic modulation of decision biases by brainstem arousal systems. Elife, 6, e23232 10.7554/eLife.23232.001 28383284 PMC5409827

[psyp13499-bib-0009] de Gee, J. W. , Knapen, T. , & Donner, T. H. (2014). Decision‐related pupil dilation reflects upcoming choice and individual bias. Proceedings of the National Academy of Sciences, 111(5), E618–E625. 10.1073/pnas.1317557111 PMC3918830 24449874

[psyp13499-bib-0010] Derksen, M. , van Alphen, J. , Schaap, S. , Mathot, S. , & Naber, M. (2018). Pupil mimicry is the result of brightness perception of the Iris and Pupil. Journal of Cognition, 1(1), 1–16. 10.5334/joc.34 PMC6634366 31517205

[psyp13499-bib-0011] Ebitz, R. B. , & Platt, M. L. (2015). Neuronal activity in primate dorsal anterior cingulate cortex signals task conflict and predicts adjustments in pupil‐linked arousal. Neuron, 85(3), 628–640. 10.1016/j.neuron.2014.12.053 25654259 PMC4319115

[psyp13499-bib-0012] Eckhoff, P. , Wong‐Lin, K. F. , & Holmes, P. (2009). Optimality and robustness of a biophysical decision‐making model under norepinephrine modulation. Journal of Neuroscience, 29(13), 4301–4311. 10.1523/JNEUROSCI.5024-08.2009 19339624 PMC2750074

[psyp13499-bib-0013] Einhäuser, W. , Stout, J. , Koch, C. , & Carter, O. (2008). Pupil dilation reflects perceptual selection and predicts subsequent stability in perceptual rivalry. Proceedings of the National Academy of Sciences, 105(5), 1704–1709. 10.1073/pnas.0707727105 PMC2234208 18250340

[psyp13499-bib-0014] Fahle, M. , Stemmler, T. , & Spang, K. M. (2011). How much of the “unconscious” is just pre ‐ threshold? Frontiers in Human Neuroscience, 5, 120 10.3389/fnhum.2011.00120 22025912 PMC3198031

[psyp13499-bib-0015] Forstmann, B. U. , Dutilh, G. , Brown, S. , Neumann, J. , Von Cramon, D. , Yves, R. , … Wagenmakers, E.‐J. (2008). Striatum and pre‐SMA facilitate decision‐making under time pressure. Proceedings of the National Academy of Sciences, 105(45), 17538–17542. 10.1073/pnas.0805903105 PMC2582260 18981414

[psyp13499-bib-0016] Gilzenrat, M. S. , Nieuwenhuis, S. , Jepma, M. , & Cohen, J. D. (2010). Pupil diameter tracks changes in control state predicted by the adaptive gain theory of locus coeruleus function. Cognitive, Affective, & Behavioral Neuroscience, 10(2), 252–269. 10.3758/CABN.10.2.252 PMC3403821 20498349

[psyp13499-bib-0017] Heitz, R. P. , & Schall, J. D. (2012). Neural mechanisms of speed‐accuracy tradeoff. Neuron, 76(3), 616–628. 10.1016/j.neuron.2012.08.030 23141072 PMC3576837

[psyp13499-bib-0018] Hess, E. H. , & Polt, J. M. (1964). Pupil size in relation to mental activity during simple problem‐solving. Science, 143(3611), 1190–1192. 10.1126/science.143.3611.1190 17833905

[psyp13499-bib-0019] Hoeks, B. , & Levelt, W. J. M. (1993). Pupillary dilation as a measure of attention: A quantitative system analysis. Behavior Research Methods, Instruments, & Computers, 25(1), 16–26. 10.3758/BF03204445

[psyp13499-bib-0020] Jepma, M. , & Nieuwenhuis, S. (2011). Pupil diameter predicts changes in the exploration‐exploitation trade‐off: Evidence for the adaptive gain theory. Journal of Cognitive Neuroscience, 23(7), 1587–1596. 10.1162/jocn.2010.21548 20666595

[psyp13499-bib-0021] Joshi, S. , Li, Y. , Kalwani, R. M. , & Gold, J. I. (2016). Relationships between pupil diameter and neuronal activity in the locus coeruleus, colliculi, and cingulate cortex. Neuron, 89(1), 221–234. 10.1016/j.neuron.2015.11.028 26711118 PMC4707070

[psyp13499-bib-0022] Kloosterman, N. A. , Meindertsma, T. , Loon, A. M. , Lamme, V. A. F. , Bonneh, Y. S. , & Donner, T. H. (2015). Pupil size tracks perceptual content and surprise. European Journal of Neuroscience, 41(8), 1068–1078. 10.1111/ejn.12859 25754528

[psyp13499-bib-0023] Koenig, S. , Uengoer, M. , & Lachnit, H. (2018). Pupil dilation indicates the coding of past prediction errors: Evidence for attentional learning theory. Psychophysiology, 55(4), e13020 10.1111/psyp.13020 29023832

[psyp13499-bib-0024] Koss, M. C. (1986). Pupillary dilation as an index of central nervous system alpha 2‐adrenoceptor activation. Journal of Pharmacological Methods, 15(1), 1–19. 10.1016/0160-5402(86)90002-1 2869190

[psyp13499-bib-0025] Krishnamurthy, K. , Nassar, M. R. , Sarode, S. , & Gold, J. I. (2017). Arousal‐related adjustments of perceptual biases optimize perception in dynamic environments. Nature Human Behaviour, 1(6), 0107 10.1038/s41562-017-0107 PMC5638136 29034334

[psyp13499-bib-0026] Laeng, B. , Sirous, S. , & Gredebäck, G. (2012). Pupillometry: A window to the preconscious? Perspectives on Psychological Science, 7(1), 18–27. 10.1177/1745691611427305 26168419

[psyp13499-bib-0027] Lavín, C. , San Martín, R. , & Rosales Jubal, E. (2014). Pupil dilation signals uncertainty and surprise in a learning gambling task. Frontiers in Behavioral Neuroscience, 7, 218 10.3389/fnbeh.2013.00218 24427126 PMC3879532

[psyp13499-bib-0028] Liu, Y. , Rodenkirch, C. , Moskowitz, N. , Schriver, B. , & Wang, Q. I. (2017). Dynamic lateralization of pupil dilation evoked by locus coeruleus activation results from sympathetic, not parasympathetic contributions. Cell Reports, 20(13), 3099–3112. 10.1016/j.celrep.2017.08.094 28954227 PMC5679481

[psyp13499-bib-0029] Mather, M. , Clewett, D. , Sakaki, M. , & Harley, C. W. (2016). Norepinephrine ignites local hotspots of neuronal excitation: How arousal amplifies selectivity in perception and memory. Behavioral and Brain Sciences, 39, e200 10.1017/S0140525X15000667 26126507 PMC5830137

[psyp13499-bib-0030] Mathôt, S. , Van der Linden, L. , Grainger, J. , & Vitu, F. (2013). The pupillary light response reveals the focus of covert visual attention. PLoS ONE, 8(10), e78168 10.1371/journal.pone.0078168. 24205144 PMC3812139

[psyp13499-bib-0031] McGinley, M. J. , David, S. V. , & McCormick, D. A. (2015). Cortical membrane potential signature of optimal states for sensory signal detection. Neuron, 87(1), 179–192. 10.1016/j.neuron.2015.05.038 26074005 PMC4631312

[psyp13499-bib-0032] Murphy, P. R. , Robertson, I. H. , Balsters, J. H. , & O'Connell, R. G. (2011). Pupillometry and P3 index the locus coeruleus–noradrenergic arousal function in humans. Psychophysiology, 48(11), 1532–1543. 10.1111/j.1469-8986.2011.01226.x 21762458

[psyp13499-bib-0033] Murphy, P. R. , O'Connell, R. G. , O'Sullivan, M. , Robertson, I. H. , & Balsters, J. H. (2014). Pupil diameter covaries with BOLD activity in human locus coeruleus. Human Brain Mapping, 35(8), 4140–4154. 10.1002/hbm.22466 24510607 PMC6869043

[psyp13499-bib-0034] Murphy, P. R. , Vandekerckhove, J. , & Nieuwenhuis, S. (2014). Pupil‐linked arousal determines variability in perceptual decision making. PLoS Computational Biology, 10(9), e1003854 10.1371/journal.pcbi.1003854 25232732 PMC4168983

[psyp13499-bib-0035] Murphy, P. R. , Boonstra, E. , & Nieuwenhuis, S. (2016). Global gain modulation generates time‐dependent urgency during perceptual choice in humans. Nature Communications, 7, 13526 10.1038/ncomms13526 PMC5123079 27882927

[psyp13499-bib-0036] Murphy, P. R. , van Moort, M. L. , & Nieuwenhuis, S. (2016). The pupillary orienting response predicts adaptive behavioral adjustment after errors. PLoS ONE, 11(3), e0151763 10.1371/journal.pone.0151763 27010472 PMC4807057

[psyp13499-bib-0037] Naber, M. , Alvarez, G. A. , & Nakayama, K. (2013). Tracking the allocation of attention using human pupillary oscillations. Frontiers in Psychology, 4(919), 10.3389/fpsyg.2013.00919 PMC3857913 24368904

[psyp13499-bib-0038] Naber, M. , Frässle, S. , & Einhäuser, W. (2011). Perceptual rivalry: Reflexes reveal the gradual nature of visual awareness. PLoS ONE, 6(6), e20910 10.1371/journal.pone.0020910 21677786 PMC3109001

[psyp13499-bib-0039] Naber, M. , Frässle, S. , Rutishauser, U. , & Einhäuser, W. (2013). Pupil size signals novelty and predicts later retrieval success for declarative memories of natural scenes. Journal of Vision, 13(2), 11 10.1167/13.2.11 23397036

[psyp13499-bib-0040] Naber, M. , Hommel, B. , & Colzato, L. S. (2015). Improved human visuomotor performance and pupil constriction after choline supplementation in a placebo‐controlled double‐blind study. Scientific Reports, 5, 13188 10.1038/srep13188 26271904 PMC4536529

[psyp13499-bib-0041] Naber, M. , & Nakayama, K. (2013). Pupil responses to high‐level image content. Journal of Vision, 13(6), 7 10.1167/13.6.7 23685390

[psyp13499-bib-0042] Nassar, M. R. , Rumsey, K. M. , Wilson, R. C. , Parikh, K. , Heasly, B. , & Gold, J. I. (2012). Rational regulation of learning dynamics by pupil‐linked arousal systems. Nature Neuroscience, 15(7), 1040–1046. 10.1038/nn.3130 22660479 PMC3386464

[psyp13499-bib-0043] Nelson, A. , & Mooney, R. (2016). The basal forebrain and motor cortex provide convergent yet distinct movement‐related inputs to the auditory cortex. Neuron, 90(3), 635–648. 10.1016/j.neuron.2016.03.031 27112494 PMC4866808

[psyp13499-bib-0044] Nieuwenhuis, S. , de Geus, E. J. , & Aston‐Jones, G. (2011). The anatomical and functional relationship between the P3 and autonomic components of the orienting response. Psychophysiology, 48, 162–175. 10.1111/j.1469-8986.2010.01057.x 20557480 PMC3797154

[psyp13499-bib-0045] Niyogi, R. K. , & Wong‐Lin, K. F. (2013). Dynamic excitatory and inhibitory gain modulation can produce flexible, robust and optimal decision‐making. PLoS Computational Biology, 9(6), e1003099 10.1371/journal.pcbi.1003099 23825935 PMC3694816

[psyp13499-bib-0046] Preuschoff, K. , t Hart, B. M. , & Einhäuser, W. (2011). Pupil dilation signals surprise: Evidence for noradrenaline’s role in decision making. Frontiers in Decision Neuroscience, 5(115), 10.3389/fnins.2011.00115 PMC3183372 21994487

[psyp13499-bib-0047] Rajkowski, J. , Kubiak, P. , & Aston‐Jones, G. (1993). Correlations between locus coeruleus (LC) neural activity, pupil diameter and behavior in monkey support a role of LC in attention. Society for Neuroscience Abstracts, 19, 974.

[psyp13499-bib-0048] Reimer, J. , McGinley, M. J. , Liu, Y. , Rodenkirch, C. , Wang, Q. I. , McCormick, D. A. , & Tolias, A. S. (2016). Pupil fluctuations track rapid changes in adrenergic and cholinergic activity in cortex. Nature Communications, 7, 13289 10.1038/ncomms13289 PMC5105162 27824036

[psyp13499-bib-0049] Schriver, B. J. , Bagdasarov, S. , & Wang, Q. I. (2018). Pupil‐linked arousal modulates behavior in rats performing a whisker deflection direction discrimination task. Journal of Neurophysiology, 120(4), 1655–1670. 10.1152/jn.00290.2018 29995602 PMC6230792

[psyp13499-bib-0050] Servan‐Schreiber, D. , Printz, H. , & Cohen, J. D. (1990). A network model of catecholamine effects: Gain, signal‐to‐noise ratio, and behavior. Science, 249(4971), 892–895. 10.1126/science.2392679 2392679

[psyp13499-bib-0051] Standage, D. , You, H. , Wang, D. H. , & Dorris, M. C. (2011). Gain modulation by an urgency signal controls the speed–accuracy trade‐off in a network model of a cortical decision circuit. Frontiers in Computational Neuroscience, 5, 7 10.3389/fncom.2011.00007 21415911 PMC3042674

[psyp13499-bib-0052] Steinhauer, S. R. , Siegle, G. J. , Condray, R. , & Pless, M. (2004). Sympathetic and parasympathetic innervation of pupillary dilation during sustained processing. International Journal of Psychophysiology, 52(1), 77–86. 10.1016/j.ijpsycho.2003.12.005 15003374

[psyp13499-bib-0053] Sterpenich, V. , D'Argembeau, A. , Desseilles, M. , Balteau, E. , Albouy, G. , Vandewalle, G. , … Maquet, P. (2006). The locus ceruleus is involved in the successful retrieval of emotional memories in humans. Journal of Neuroscience, 26(28), 7416–7423. 10.1523/JNEUROSCI.1001-06.2006 16837589 PMC6674193

[psyp13499-bib-0054] Thura, D. , & Cisek, P. (2016). Modulation of premotor and primary motor cortical activity during volitional adjustments of speed‐accuracy trade‐offs. Journal of Neuroscience, 36(3), 938–956. 10.1523/JNEUROSCI.2230-15.2016 26791222 PMC6602002

[psyp13499-bib-0055] van den Brink, R. L. , Murphy, P. R. , & Nieuwenhuis, S. (2016). Pupil diameter tracks lapses of attention. PLoS ONE, 11(10), e0165274 10.1371/journal.pone.0165274 27768778 PMC5074493

[psyp13499-bib-0056] Van Steenbergen, H. , & Band, G. P. H. (2013). Pupil dilation in the Simon task as a marker of conflict processing. Frontiers in Human Neuroscience, 7, 215 10.3389/fnhum.2013.00215 23754997 PMC3665936

[psyp13499-bib-0057] Varazzani, C. , San‐Galli, A. , Gilardeau, S. , & Bouret, S. (2015). Noradrenaline and dopamine neurons in the reward/effort trade‐off: A direct electrophysiological comparison in behaving monkeys. Journal of Neuroscience, 35(20), 7866–7877. 10.1523/JNEUROSCI.0454-15.2015 25995472 PMC6795183

[psyp13499-bib-0058] Vaughan, H. G. Jr , Costa, L. D. , & Gilden, L. (1966). The functional relation of visual evoked response and reaction time to stimulus intensity. Vision Research, 6(11–12), 645–656. 10.1016/0042-6989(66)90076-9 6003387

[psyp13499-bib-0059] Vinck, M. , Batista‐Brito, R. , Knoblich, U. , & Cardin, J. A. (2015). Arousal and locomotion make distinct contributions to cortical activity patterns and visual encoding. Neuron, 86(3), 740–754. 10.1016/j.neuron.2015.03.028 25892300 PMC4425590

[psyp13499-bib-0060] Wang, Y. , Zekveld, A. A. , Naylor, G. , Ohlenforst, B. , Jansma, E. P. , Lorens, A. , … Kramer, S. E. (2016). Parasympathetic nervous system dysfunction, as identified by pupil light reflex, and its possible connection to hearing impairment. PLoS ONE, 11(4), e0153566 10.1371/journal.pone.0153566 27089436 PMC4835104

[psyp13499-bib-0061] Warren, C. M. , Eldar, E. , van den Brink, R. L. , Tona, K.‐D. , van der Wee, N. J. , Giltay, E. J. , … Nieuwenhuis, S. (2016). Catecholamine‐mediated increases in gain enhance the precision of cortical representations. Journal of Neuroscience, 36(21), 5699–5708. 10.1523/JNEUROSCI.3475-15.2016 27225761 PMC6601838

[psyp13499-bib-0062] Wessel, J. R. , Danielmeier, C. , & Ullsperger, M. (2011). Error awareness revisited: Accumulation of multimodal evidence from central and autonomic nervous systems. Journal of Cognitive Neuroscience, 23(10), 3021–3036. 10.1162/jocn.2011.21635 21268673

[psyp13499-bib-0063] Wierda, S. M. , van Rijn, H. , Taatgen, N. A. , & Martens, S. (2012). Pupil dilation deconvolution reveals the dynamics of attention at high temporal resolution. Proceedings of the National Academy of Sciences, 109(22), 8456–8460. 10.1073/pnas.1201858109 PMC3365158 22586101

[psyp13499-bib-0064] Woodhouse, J. M. , & Campbell, F. W. (1975). The role of the pupil light reflex in aiding adaptation to the dark. Vision Research, 15(6), 649–653. 10.1016/0042-6989(75)90279-5 1138479

[psyp13499-bib-0065] Woodworth, R. S. (1899). Accuracy of voluntary movement. The Psychological Review: Monograph Supplements, 3(3), i. 10.1037/h0092992

[psyp13499-bib-0066] Yerkes, R. M. , & Dodson, J. D. (1908). The relation of strength of stimulus to rapidity of habit‐formation. Journal of Comparative Neurology and Psychology, 18(5), 459–482. 10.1002/cne.920180503

