# Supplementary material for: Pupillometric investigation into the speed‐accuracy trade‐off in a visuo‐motor aiming task
Source: Psychophysiology. 2019 Nov 17;57(3):e13499. doi: 10.1111/psyp.13499 (PMC7027463; doi:10.1111/psyp.13499)
Supplement: Supplementary file 1 — Figure S1 Behavioral performance per binned percentile pupil size. Means and standard errors are shown. [file PSYP-57-e13499-s001.docx]

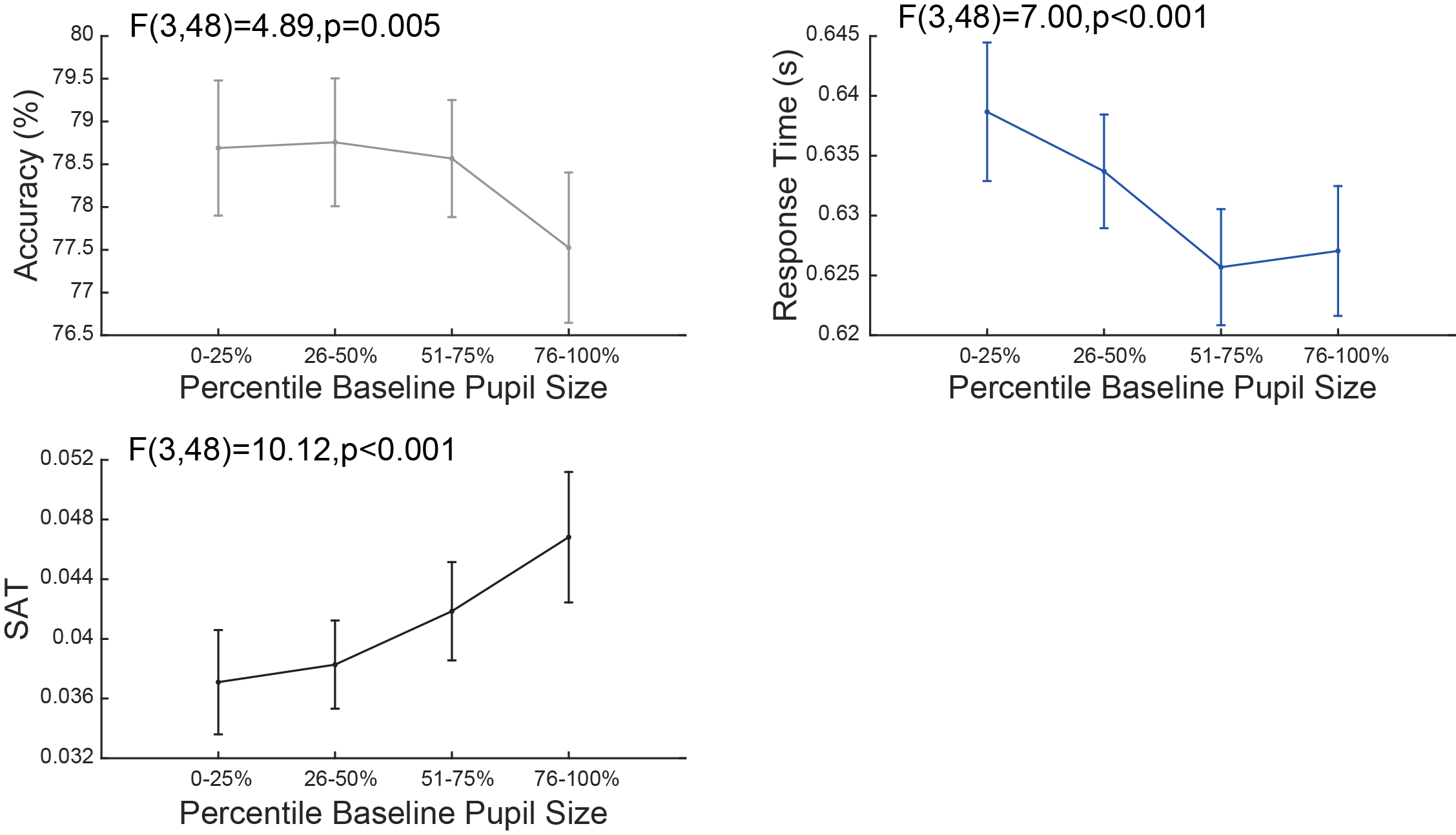


**Figure S1. Behavioral performance per binned percentile pupil size.** Means and standard errors are shown.
