# Supplementary material for: Pupillometric investigation into the speed‐accuracy trade‐off in a visuo‐motor aiming task
Source: Psychophysiology. 2019 Nov 17;57(3):e13499. doi: 10.1111/psyp.13499 (PMC7027463; doi:10.1111/psyp.13499)
Supplement: Supplementary file 2 — Table S1 Behavioral performance per speed/accuracy instruction. Means and standard deviations are shown per behavioral performance measures and per instruction. [file PSYP-57-e13499-s002.docx]

| **Measure** | **Fast** | **Accurate** | **Trade-off** |
| --- | --- | --- | --- |
| Response Times | 0.62s ± 0.03s | 0.64s ± 0.02s | 0.63s ± 0.02s |
| Accuracy | 77% ± 3% | 79% ± 3% | 79% ± 3% |

**Table S1. Behavioral performance per speed/accuracy instruction.** Means and standard deviations are shown per behavioral performance measures and per instruction.
